# Low-Dose Gemcitabine Treatment Enhances Immunogenicity and Natural Killer Cell-Driven Tumor Immunity in Lung Cancer

**DOI:** 10.3389/fimmu.2020.00331

**Published:** 2020-02-25

**Authors:** Xin Zhang, Dong Wang, Zhidong Li, Defeng Jiao, Linlin Jin, Jingjing Cong, Xiaohu Zheng, Lijun Xu

**Affiliations:** ^1^Department of Respiratory Medicine, The First Hospital of Jilin University, Changchun, China; ^2^Hefei National Laboratory for Physical Sciences at Microscale, The CAS Key Laboratory of Innate Immunity and Chronic Disease, Division of Molecular Medicine, School of Life Sciences, University of Science and Technology of China, Hefei, China; ^3^Department of Rehabilitation Medicine, The First Hospital of Jilin University, Changchun, China

**Keywords:** low-dose gemcitabine, NK cells, immunogenicity, antitumor immunity, lung cancer

## Abstract

Gemcitabine has been used as first-line chemotherapy against lung cancer, but many patients experience cancer recurrence. Activation of anti-tumor immunity *in vivo* has become an important way to prevent recurrence. Anti-tumor immune responses are often dependent upon the immunogenicity of tumors. In our study, we observed that low-dose gemcitabine treatment enhanced the immunogenicity of lung cancer by increasing the exposure of calreticulin, high mobility group box 1, and upregulating expression of NKG2D ligands. Further studies demonstrated that low-dose gemcitabine treatment increased interferon-γ expression and NK-cell activation in mice. Low-dose gemcitabine treatment was sufficient for inhibiting tumor growth with few side effects *in vivo*. These data suggest that low-dose gemcitabine-induced immunochemotherapy activated antitumor immunity in immunocompetent patients.

## Introduction

Lung cancer is associated with high morbidity and mortality. Gemcitabine is a nucleoside analog that inhibits DNA synthesis. Gemcitabine is a first-line chemotherapy against lung cancer (1, 2). Although gemcitabine can kill cancer cells effectively, many patients suffer from cancer recurrence.

Activation of anti-tumor immunity has become an efficacious way to reduce the risk of cancer recurrence. Recent studies have suggested that cytotoxic chemotherapy also has several immune mechanisms (3). Hence, the most efficacious strategy to prevent cancer recurrence may be to mobilize and stimulate the immune system to act against tumor cells.

The immunogenicity of cancer cells is an emerging determinant of anti-cancer immunotherapy (4). Research has shown that, upon certain anticancer treatments, dying cancer cells emit immunomodulatory factors, or damage-associated molecular patterns (DAMPs). These serve as “danger signals” and enhance the immunogenicity of cancer cells against anticancer immune responses (5). DAMPs are endogenous molecules that are secreted, released or surface-exposed by dying, stressed, or injured cells to mediate robust immunomodulatory effects. DAMPs combine with pattern recognition sensors to produce powerful stimulation of the immune system. Most of these molecules have non-immunological functions within cells before their exposure or release (6–8).

High-mobility group box 1 protein (HMGB1) is a DAMP. It is a non-histone chromatin-binding protein and a potential way of inducing an immune response against dying tumor cells undergoing necrosis (9). Calreticulin (CRT), as an “eat me” signal, is elicited by some cytotoxic chemotherapy. CRT can induce membrane expression, and membranes become receptive to engulfment by dendritic cells (DCs) (10, 11). In addition, some DAMPs are vital for recognition and elimination of tumor cells by natural killer (NK) cells, including NKG2D ligands such as major histocompatibility complex class I polypeptide-related sequence A (MICA), major histocompatibility complex class I polypeptide-related sequence B (MICB), retinoic acid early transcript (RAET)1E, and the UL16 binding protein (ULBP) family (12, 13). NKG2D ligands are expressed mostly on tumor cells but show low expression on the surface of healthy cells so that immune cells are not activated (14). It has been reported that tumor cells with high expression of NKG2D ligands are rejected efficiently by NK cells, but that tumor cells with intermediate expression of NKG2D ligands are less immunogenic (15). Consequently, increasing expression of NKG2D ligands on tumor cells and thus increasing the killing activity of NK cells could enhance anti-tumor immunity effectively.

Certain types of chemotherapy help tumor cells to express DAMPs, which interact with the immune system and induce immunogenicity (10, 16). Morisaki et al. showed that gemcitabine-induced effects on MICA/B expression enhance the susceptibility of HepG2 hepatocellular carcinoma cells to the cytotoxic actions of cytokine-activated killer (CAK) cells (17). It has also been found that low-dose gemcitabine can induce MICA/B expression on some pancreatic cancer cell lines (18). We wanted to ascertain if gemcitabine can activate antitumor immunity and, if so, its mechanism of action. Here, we studied the immunogenicity of gemcitabine *in vitro* and established a model of lung cancer in mice. *In vitro*, we observed that low-dose gemcitabine treatment enhanced the immunogenicity by increasing the exposure of CRT, HMGB1, and upregulating expression of NKG2D ligands. The latter were treated with a high (120 mg/kg body weight), medium (60 mg/kg body weight), or low (30 mg/kg body weight) dose of gemcitabine *in vivo*. We found that a low dose of gemcitabine was effective in inducing CRT and HMGB1 exposure, aiding NK cells to kill tumors.

## Materials and Methods

### Ethical Approval of the Study Protocol

All procedures involving experimental animals were in accordance with the National Guidelines for Animal Usage in Research for China. The study protocol was approved by the Ethics Committee of the University of Science and Technology of China (Hefei, China).

### Cell Lines

The murine Lewis lung carcinoma (LLC) cell line and human lung cancer cell (A549) line were purchased from the Cell Bank of the Chinese Academy of Sciences (Shanghai, China). LLC cells were maintained in Dulbecco's modified Eagle's medium (DMEM; HyClone, USA). A549 cells were cultured in Roswell Park Memorial Institute (RPMI) 1640 medium (HyClone, USA). Both cell lines were supplemented with 10% fetal bovine serum (Gibco, Billings, MT, USA) and 1% penicillin/streptomycin (Beyotime Bio, Jiangsu, China) and maintained at 37°C in a 5% CO_2_ incubator. Cells at logarithmic growth were used for *in vitro* and *in vivo* experiments.

### Mice

Male C57BL/6 mice were purchased form Charles River Laboratories (Beijing, China) and used at 6–8 weeks of age. Mice were fed under specific pathogen-free conditions and had free access to water and a standard rodent diet.

### *In vivo* Experiments

To establish a tumor model of LLC, LLC cells (10^6^) in 100 μL of phosphate-buffered saline (PBS) were inoculated (s.c.) on the right flank of C57BL/6 mice. Chemotherapy was started when tumors reached 50–150 mm^3^.

Before treatment, mice were randomized into four groups of five. One group receiving PBS served as the control group. The other three groups were injected (i.p.) with gemcitabine (#S1714; Selleck Chemicals, Houston, TX, USA) at 120, 60, or 30 mg/kg (four times every 3 days) plus cisplatin (#S1166; Selleck Chemicals) at 3 mg/kg (twice every 6 days). Tumor size (0.5 × length × width^2^) was measured by an electronic caliper twice a day. Body weight were monitored on an electronic scale every other day. Biological tissue was harvested from mice after treatment.

### Cell-Surface CRT Expression and Nuclear HMGB1 Exposure

LLC cells and A549 cells were cultured on 24-well plates (2 × 10^5^/well) overnight. Then, cells were treated for 24 or 48 h with gemcitabine (Gem) (5, 10, 50, 100, or 500 nM), cisplatin (CDDP) (5 μM), or mitoxantrone (Mtx) (1 μM). Tumor tissues were frozen by Optimum Cutting Temperature formulation (Sakura Finetek, Torrance, CA, USA). Cells were fixed with 4% paraformaldehyde for 15 min and washed in PBS. For surface detection of CRT, cells and tissues were stained with a rabbit anti-calreticulin antibody (ab2907; Abcam, Cambridge, UK) overnight. After three washes in PBS, cells or tissues were incubated for 1 h with Alexa Flour 546-conjugated secondary antibody to rabbit IgG (Invitrogen, Carlsbad, CA, USA). For nuclear HMGB1 exposure, cells and tissues were fixed with 4% paraformaldehyde for 15 min and then incubated in 0.3% Triton-X 100 (Solarbio, Beijing, China) for 15 min. After washing in PBS, cells and tissues were incubated overnight with anti-HMGB1 antibody (ab195011; Abcam). All samples were stained with 4′,6-diamidino-2-phenylindole (D3571; Invitrogen) for 5 min at room temperature. A multi-photon confocal microscope (880 Meta; Zeiss, Weltzlar, Germany) was used to measure immunofluorescence.

### Quantitative Real-Time Polymerase Chain Reaction (qRT-PCR)

LLC cells and A549 cells were cultured on 6-well plates overnight. Then, cells were treated with vehicle control (DMSO), gemcitabine (Gem) (10 nM), cisplatin (CDDP) 5 μM. RNA samples were isolated using TRIzol® Reagent (Invitrogen). cDNA synthesis was done using M-MLV Reverse Transcriptase (Invitrogen) and random primers (Invitrogen). qRT-PCR was carried out using TB Green Premix Ex Taq *II* (TaKaRa Biotechnology, Shiga, Japan). The primers used in this study are shown in Table 1. For mice, all target genes were compared with *Actin*. For humans, all target genes were compared with *GAPDH*.

**Table 1:** List of primers used in qRT-PCR.

**Name of Gene**	**Primers** **(5^′^ → 3^′^)**	**Source**
*H60*	Forward: ACACTTTAGAGAAATGCCAAAATCA	NCBI
	Reverse: TCCAGGTGAAACTCAGACCC	
*Raet-1*	Forward: TCCGCAAAGCCAGGGCCAAA	(19)
	Reverse: GCTGGTAGGTGGAAGCGGGG	
*Ulbp1*	Forward: CTAACACAACCGGAAAGCCCCT	(19)
	Reverse: CAGTGCTTGTGTCAACACGGA	
*Actin*	Forward: CCACTGTCGAGTCGCGTCC	NCBI
	Reverse: ATTCCCACCATCACACCCTGG	
*MICA*	Forward: CCTTGGCCATGAACGTCAGG	(20)
	Reverse: CCTCTGAGGCCTCGCTGCG	
*MICB*	Forward: ACCTTGGCTATGAACGTCACA	(20)
	Reverse: CCCTCTGAGACCTCGCTGCA	
*ULBP1*	Forward: CAAGTGGAGAATTTAATACCCATTGAG	(20)
	Reverse: TGTTGTTTGAGTCAAAGAGGA	
*ULBP2*	Forward: TTACTTCTCAATGGGAGACTGT	(20)
	Reverse: TGTGCCTGAGGACATGGCGA	
*ULBP3*	Forward: AGGTCTTATCTATGGGTCACCTAGAAG	(21)
	Reverse: CTGAAATCCTCCAGCTCAGTGTCAG	
*ULBP4*	Forward: CCTCAGGATGCTCCTTTGTGA	(20)
	Reverse: CGACTTGCAGAGTGGAAGGATC	
*ULBP5*	Forward: TGGCCGACCCTCACTCTCT	(20)
	Reverse: CCGTGGTCCAGGTCTGAACT	
*ULBP6*	Forward: AATCTCTTGTCCCCAGCCCTC	NCBI
	Reverse: TTCATCCACCTGGCCTTGAA	
*GAPDH*	Forward: GAAGGTGAAGGTCGGAGTC	(22)
	Reverse: GAAGATGGTGATGGGATTTC	

### Blood Collection

Blood of the mice was collected in heparin anticoagulation tubes. Blood components were detected by a hematology analyzer (XT-1800i; Sysmex, Tokyo, Japan).

### Flow Cytometry

The antibodies used for flow cytometry were purchased from Biolegend [San Diego, CA, USA; fluorescein isothiocyanate (FITC)-labeled cluster of differentiation (CD)8α (5H10-1), Percp-CY5.5-labeled interferon (IFN)-γ (XMG1.2), CD8α (53–6.7), BV421-labeled interferon (IFN)-γ (XMG1.2), PE-CY7-labeled NK1.1 (PK136), APC-CY7-labeled CD45.2 (104), BV510-labeled CD3e (145-2C11)], eBioscience [San Diego, CA, USA; APC-labeled NKG2D (CX5)] or from BD Biosciences [Franklin Lakes, NJ, USA; FITC-labeled CD19 (1D3), PE-labeled CD3 (145-2C11), BV510-labeled Ki67 (B56), BV605-labeled CD3 (145-2C11), BV786-labeled CD4 (GK1.5)].

For extracellular staining, 5% normal rat serum was added to isolated lymphocytes from mouse spleens for 30 min at 4°C (to block Fc receptors) followed by incubation with extracellular antibodies for 30 min at 4°C. For intracellular staining, freshly isolated spleen lymphocytes from mice were incubated with phorbol myristate acetate (50 ng/mL; Sigma–Aldrich, Saint Louis, MO, USA), monensin (10 μg/mL; Sigma–Aldrich) and ionomycin (1 μg/mL; Sigma–Aldrich) for 4 h at 37°C in a 5% CO_2_ incubator. Then, lymphocytes were incubated with 5% normal rat serum (to block Fc receptors) and stained with extracellular antibodies for 30 min at 4°C. After fixation and permeabilization, lymphocytes were stained with intracellular antibodies for 30 min at 4°C. Flow cytometry was carried out using an LSRIIsystem (BD Biosciences) and analyzed with FlowJo (Tree Star, Ashland, OR, USA).

### Histology

The livers and kidneys of mice were fixed in 12% neutral buffered formalin for 5 days, and then dehydrated and embedded in paraffin. Sections of liver and kidney tissues were cut were into 5 μm-thick sections and stained with hematoxylin and eosin (H&E).

### Isolation of Lymphocytes and NK Cells From Spleens

Mouse spleens were ground and filtered through sieves. Erythrocytes were lysed at room temperature with erythrocyte lysate for 5 min. NK cells were purified with biotin-NKp46 antibodies and anti-biotin microbeads (29A1.4; Biolegend) and enriched by magnetic cell sorting (MACS) columns (Miltenyi Biotec, Bergisch Gladbach, Germany). Spleen NKp46^+^ cells were incubated with Percp-CY 5.5-labeled-CD3 (Biolegend) fluorescent Abs, and a FACSAriaIICell Sorter (San Jose, CA, USA) was used to purify. The purity was above 90%.

### Carboxyfluorescein Succinimidyl Ester (CFSE) Assay

A mixture of purified NK cells and CFSE-labeled (21888; Sigma–Aldrich) LLC cells or gemcitabine (10 nM)-treated LLC cells was plated in triplicate in a 96-well plate. NK cells and target cells were at an effector cell:target cell ratio of 1:1, 4:1, and 10:1. Cells were incubated at 37°C for 6 h, and then 7AAD (559925; BD Biosciences) was added before detection by flow cytometry.

### Real-Time Cellular Analysis (RTCA)

Before seeding LLC cells, 50 μL of DMEM containing 10% fetal bovine serum was added to each well of E-Plates for measurement of impedance background. Then, LLC cells (2 × 10^5^/mL) were added to the E-Plate (50 μL/well) on a clean bench for 30 min. E-Plates were incubated at 37°C in a 5% CO_2_ incubator and monitored on a RTCA system at 15-min intervals. Purified NK cells were added to the culture medium at an effector:target ratio of 10:1 and 20:1 when LLC cells were in logarithmic growth. The cell index was a parameter that reflected cellular proliferation based on the impedance measurement obtained using the RTCA system. The “normalized time” was a manipulation of the data (i.e., the last measurement time point before addition of purified NK cells), which was then set as 1.0 by the xCELLigence v2.1.

### mAb Reagents and Flow Cytometry for Expression of NKG2D Ligands

A549 cells were cultured in 12-well culture plates (3 × 10^5^/well) overnight. Then, cells were treated for 24 h with vehicle control (DMSO), gemcitabine or cisplatin. Expression of NKG2D ligands on A549 cells was analyzed by flow cytometry using anti-MICA/B (BD Biosciences), anti-ULBP1, anti-ULBP2/5/6 and anti-ULBP3 antibody. Anti-human ULBP1, ULBP2/5/6, and ULBP3 antibody were purchased from R&D Systems. Flow cytometry was carried out using an LSRIIsystem (BD Biosciences) and analyzed with FlowJo.

### Dead Cell Staining

LLC cells and A549 cells were cultured on 96-well plates (5 × 10^3^/well) overnight. Then, cells were treated with vehicle control (DMSO), gemcitabine (Gem; 5, 10, 50, 100, 500 nM), mitoxantrone (Mtx; 1 μM) or cisplatin (CDDP; 5 μM) at 37°C in a 5% CO2 incubator. Cells were stained with DEAD staining (R37601, Invitrogen™) for 15 min. A multi-photon confocal microscope (880 Meta; Zeiss, Weltzlar, Germany) was used to measure immunofluorescence.

### Statistical Analyses

Data are the mean ± SEM. Prism 6.0 (GraphPad, San Diego, CA, USA) was used to analyze unpaired two-tailed *t*-tests or analysis of variance (ANOVA). *P* < 0.05 was considered significant.

## Results

### Low-Dose Gemcitabine Enhances Exposure of Immunogenic Molecules in Lung Cancer

**Figure 1:**
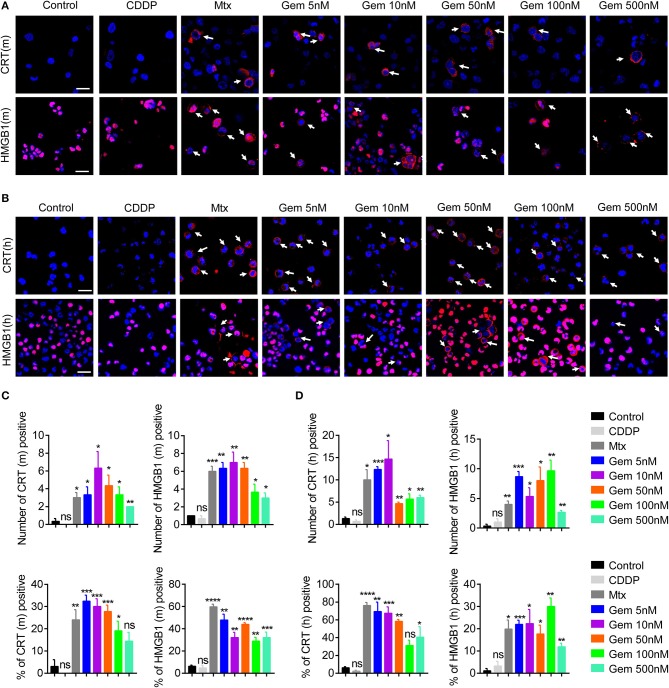
Low-dose gemcitabine treatment enhances exposure of immunogenic molecules in lung cancer *in vitro*. **(A,B)** Immunofluorescence analyses of calreticulin (CRT) exposure (red) after 24 h of chemotherapy and high-mobility group box 1 protein (HMGB1) exposure (red) after 48 h of chemotherapy on the murine Lewis lung carcinoma (LLC) cell line **(A)** and human lung cancer (A549) cell line **(B)**. LLC cells were treated with vehicle control [dimethyl sulfoxide (DMSO)], gemcitabine (Gem; 5, 10, 50, 100, 500 nM), mitoxantrone (Mtx; 1 μM), or cisplatin (CDDP; 5 μM). Scale bar = 20 μm. **(C,D)** We counted the number of fluorescent cells showing CRT and HMGB1 expression. The number of cells per field of view and the percentage of positive cells per field of view are shown. Data are representative of three independent experiments. The unpaired Student's *t*-test was used. *p < 0.05, **p < 0.01, ***p < 0.001, ****p < 0.0001.

Some chemotherapy drugs enhance the immune response by inducing immunogenicity, which can increase immune recognition of tumor cells (23–25). To investigate the immunogenicity of gemcitabine, CRT expression from cell-surface membranes and nuclear HMGB1 exposure were investigated by an immunofluorescence assay. LLC cells were treated with gemcitabine (5, 10, 50, 100, or 500 nM) to measure CRT and HMGB1 exposure *in vitro*. Mitoxantrone served as a positive control, and cisplatin as a control (10, 26). Immunofluorescence results suggested that CRT and HMGB1 exposure were increased after gemcitabine treatment (Figure 1A). Gemcitabine at the low concentration tested had strong CRT and HMGB1 exposure (Figure 1C). Furthermore, the same method was used to detect A549 cells: similarly, CRT and HMGB1 exposure were enhanced (Figures 1B,D). Low-dose gemcitabine treatment led to more fluorescent cells (Figure 1D). We also observed the death of many tumor cells with high-dose gemcitabine (Supplementary Figures 1A,B).

These results suggested that treatment with low-dose gemcitabine could enhance exposure to immunogenic molecules in lung cancer cells *in vitro*.

**Figure 2:**
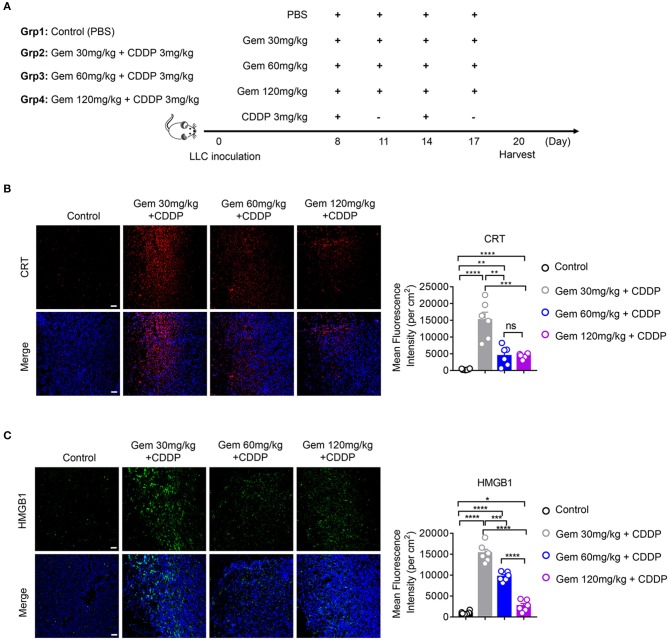
Low-dose gemcitabine treatment enhances exposure of immunogenic molecules in lung cancer *in vivo*. **(A)** C57BL/6 mice (6–8 weeks) were inoculated with 10^6^ LLC cells. Mice were divided into four groups: PBS served as a vehicle control group. The other three groups were injected (i.p.) with gemcitabine (120, 60, or 30 mg/kg, four times every 3 days) plus cisplatin (3 mg/kg, twice every 6 days). **(B,C)** Calreticulin (CRT) membrane exposure and high-mobility group box 1 protein (HMGB1) release were detected by immunofluorescence in frozen sections of subcutaneous tumors. The low-dose treatment group showed stronger mean fluorescence intensity (MFI) per square centimeter of CRT and HMGB1 than medium- or high-dose groups. Scale bar = 50 μm. Data are representative of three independent experiments. The unpaired Student's *t*-test was used. *p < 0.05, **p < 0.01, ***p < 0.001, ****p < 0.0001.

To explore if low-dose gemcitabine could enhance immunogen exposure *in vivo*, we established an LLC subcutaneous tumor model in mice. LLC cells (10^6^) in 100 μL of PBS were inoculated (s.c.) on the right flank of C57BL/6 mice. A combination of gemcitabine and cisplatin is a basic chemotherapy regimen for treatment of lung cancer. When tumors reached 50–150 mm^3^, a low (30 mg/kg), medium (60 mg/kg), or high (120 mg/kg) dose of gemcitabine combined with cisplatin (3 mg/kg) was administered. Gemcitabine was injected (i.p.) four times every 3 days and cisplatin was injected (i.p.) twice every 6 days. PBS was injected (i.p.) as a control (Figure 2A). In accordance with *in vitro* results, CRT expression in cell membranes was upregulated after gemcitabine treatment. The mean fluorescence intensity (MFI) per square centimeter of CRT was highest after low-dose (30 mg/kg) gemcitabine treatment (Figure 2B). HMGB1 exposure was augmented after gemcitabine treatment at different doses. The MFI per square centimeter of HMGB1 was highest after low-dose (30 mg/kg) gemcitabine treatment and lowest after high-dose (120 mg/kg) gemcitabine treatment (Figure 2C).

Taken together, these data suggested that low-dose gemcitabine treatment enhanced exposure of immunogenic molecules.

### Gemcitabine Induces Upregulation of Innate Immune System-Related Immunogenic Molecules in Lung Cancer

**Figure 3:**
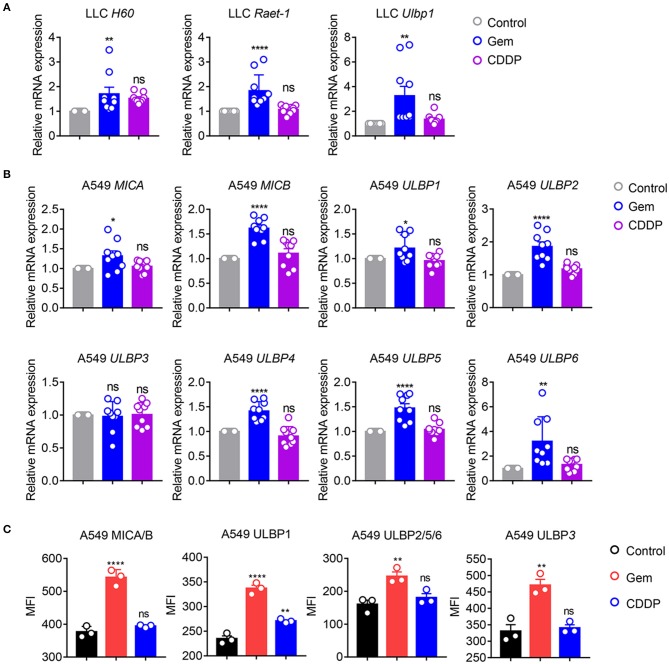
Gemcitabine up-regulates NKG2D ligands in lung cancer cells. **(A)** qRT-PCR of *H60, Raet-1*, and *Ulbp1* mRNA in LLC cells. **(B)** qRT-PCR of major histocompatibility complex class I polypeptide-related sequence A (*MICA*), *MICB*, and UL16 binding protein (*ULBP*)*1-6* mRNA in A549 cells. Data are the fold-change of mRNA expression in gemcitabine- and cisplatin-treated cells relative to untreated cells. **(C)** Surface expression of MICA/B, ULBP1, ULBP2/5/6, and ULBP3 was measured by flow cytometry on A549 cells treated with vehicle control (DMSO), gemcitabine or cisplatin. Data are representative of three independent experiments. One-way analysis of variance (ANOVA) was used. *p < 0.05, **p < 0.01, ****p < 0.0001.

NKG2D ligands, as natural immune-related molecules, are vital for recognition of tumor cells and elimination by NK cells (27–30). MICA and MICB molecules were the first NKG2D ligands to be described (27). Cellular stress induces upregulation of NKG2DLs to activate NKG2D signaling, and induces cytolytic and cytokine responses by NK cells and/or CD8^+^ T cells (28–30). To investigate if gemcitabine induced upregulation of expression of NKG2D ligands, mRNA expression of *H60, Raet-1*, and *Ulbp1* in LLC cells was measured. mRNA levels of *H60, Raet-1* and *Ulbp1* were upregulated in cells treated with gemcitabine (Figure 3A), indicating that chemotherapeutic stimulation induced upregulation of expression of NKG2D ligands in the lung cancer cells of mice. Furthermore, we investigated if expression of NKG2D ligands (MICA, MICB, ULBP1-6) was increased in A549 cells. Expression of *MICA, MICB, ULBP1, ULBP2, ULBP4, ULBP5*, and *ULBP6* were upregulated in cells treated with gemcitabine (Figure 3B). However, expression of NKG2D ligands did not change significantly in cisplatin-treated cells (Figures 3A,B). We also measured expression of human NKG2D ligands in protein levels of gemcitabine- and cisplatin-treated A549 cells by flow cytometry. We found that gemcitabine induces NKG2D ligands expression at the protein level (Figure 3C). Taken together, these data suggested that gemcitabine could induce upregulation of expression of NKG2D ligands in human and mouse lung cancer cells, thereby increasing the anti-tumor immunity of NK cells.

### Low-Dose Gemcitabine Induces Activation of NK Cells in a Mouse Model of Lung Cancer

**Figure 4:**
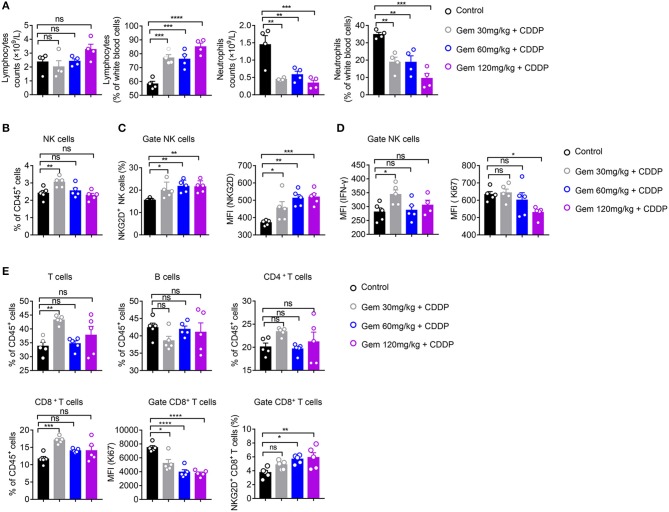
Low-dose gemcitabine induces activation of NK cells in a mouse model of lung cancer. **(A–E)** C57BL/6 mice (6–8 weeks) were inoculated with 10^6^ LLC cells. Mice were divided into four groups: PBS served as a vehicle control group. The other three groups were injected (i.p.) with gemcitabine (120, 60, or 30 mg/kg, four times every 3 days) plus cisplatin (3 mg/kg, twice every 6 days). **(A)** Percentage and absolute number of lymphocytes and neutrophils in white blood cells measured by a hematology analyzer. **(B)** The proportion (%) of NK cells in CD45^+^ cells. **(C)** Proportion of splenic NKG2D^+^ NK cells and mean fluorescence intensity (MFI) of splenic NKG2D^+^ NK cells. **(D)** MFI of splenic IFN-γ^+^ NK^+^ cells and Ki67^+^ NK^+^ cells. **(E)** Proportion of splenic T cells, B cells, CD4^+^ T cells, and CD8^+^T cells. MFI of splenic Ki67^+^ CD8^+^ T cells. The proportion (%) of splenic NKG2D^+^ CD8^+^ T cells. Data are representative of three independent experiments. One-way analysis of variance (ANOVA) was used. *p < 0.05, **p < 0.01, ***p < 0.001, ****p < 0.0001.

We further analyzed the effects of different doses of gemcitabine on the types and functions of immune cells. Compared with the control group, there was no significant change in the absolute number of lymphocytes in low (30 mg/kg)-, medium (60 mg/kg)- and high (120 mg/kg)-dose gemcitabine-treated mice, but the lymphocyte percentage was increased (Figure 4A). In all chemotherapy treatment groups, the absolute number and percentage of neutrophils declined (Figure 4A). These findings suggest that gemcitabine hampered the number of myeloid cells but not the number of lymphocytes. Therefore, we focused on changes in the number of lymphocytes. The number of NK cells increased at low-dose, but not in the medium- or high-dose, gemcitabine groups (Figure 4B, Supplementary Figure 2B). Activation of the NKG2D ligands–NKG2D pathway helps NK cells to kill tumor cells. Interestingly, gemcitabine treatment upregulated NKG2D expression in NK cells in mice (Figure 4C, Supplementary Figures 2C,D). We also found that the level of IFN-γ secretion by NK cells expressing differing levels of NKG2D is different. The level of IFN-γ produced by NKG2D^+^ NK cells is higher than NKG2D^−^ NK cells (Supplementary Figures 3A,B). Functional IFN-γ expression increased in NK cells under low-dose gemcitabine treatment (Figure 4D, Supplementary Figure 5). To evaluate the proliferation potential of NK cells, we measured the MFI of Ki67 in NK cells. We found that the MFI and percentage of Ki67 decreased in the high-dose gemcitabine group relative to that in the control group (Figure 4D, Supplementary Figures 2C, 5). The gating strategy of NK cells, B cells, CD4^+^ T cells, and CD8^+^ T cells are provided in Supplementary Figure 2A.

These data suggested that low-dose gemcitabine treatment activated the NK cell-based immune system, whereas high-dose gemcitabine treatment impaired NK-cell proliferation.

The ratio of CD8^+^ T cells of CD45^+^ cells in splenic lymphocytes increased only in the low-dose gemcitabine group. Significant differences were not observed for the number of CD4^+^ T cells and B cells in any group. All doses of gemcitabine induced an MFI decrease of Ki67^+^ CD8^+^ T cells compared with that in the control group (Figure 4E).

Taken together, these data suggested that low-dose gemcitabine treatment enhanced the NK cell-based immune response in a mouse model of lung cancer.

### Low-Dose Gemcitabine Treated Lung Cancer Cells Are More Sensitive to NK Cell Killing

**Figure 5:**
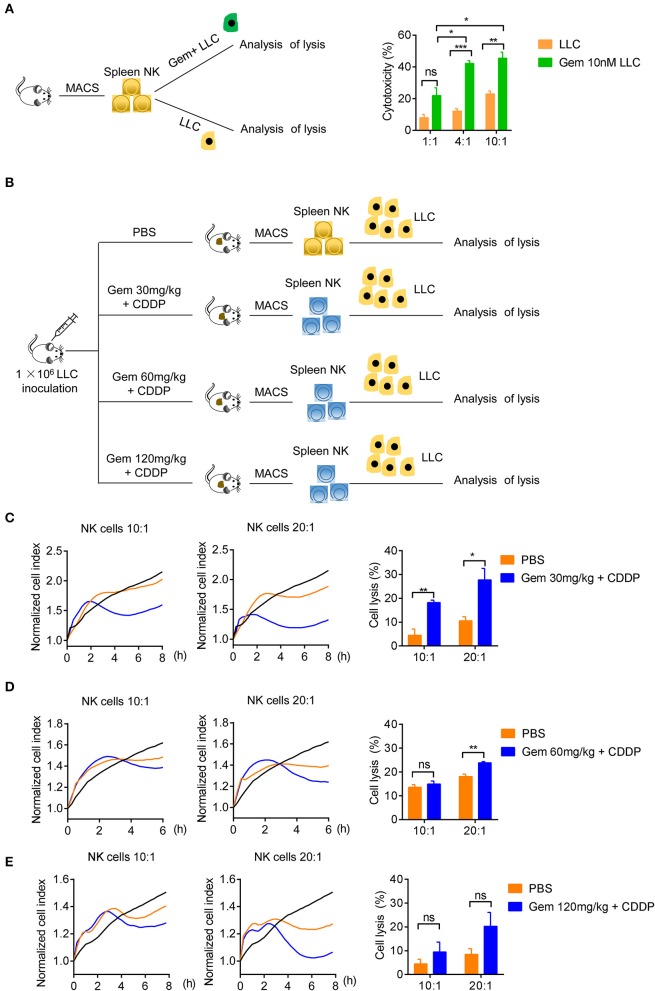
Low-dose gemcitabine treated lung cancer cells are more sensitive to NK cell killing. **(A)** Lysis of purified spleen NK cells against LLC cells or gemcitabine (10 nM)-treated LLC cells (24 h) at an effector cell:target cell ratio of 1:1, 4:1, and 10:1. **(B)** Purified NK cells of subcutaneous tumor-burdened mice injected (i.p.) with PBS (control) or gemcitabine (120, 60, or 30 mg/kg, four times every 3 days) plus cisplatin (3 mg/kg, twice every 6 days) killed LLC cells. **(C)** Purified splenic NK cells of gemcitabine (30 mg/kg) treatment subcutaneous tumor-burdened mice group killed LLC cells at an effector cell:target cell ratio of 10:1 and 20:1. **(D)** Purified splenic NK cells of gemcitabine (60 mg/kg) treatment subcutaneous tumor-burdened mice group killed LLC cells at an effector cell:target cell ratio of 10:1 and 20:1. **(E)** Purified splenic NK cells of gemcitabine (120 mg/kg) treatment subcutaneous tumor-burdened mice group killed LLC cells at an effector cell:target cell ratio of 10:1 and 20:1. **(C–E)** Splenic NK cells were purified by a FACSAria II Cell Sorter (San Jose, CA, USA) and dead cells were eliminated. The tumor cells (LLC cells) were not treated with chemotherapy drugs. The vertical line of left panels is the normalized cell index. This indicates the normalized time (i.e., the measurement time point before addition of purified NK cells), which was then set as 1.0 by xCELLigence v2.1. There were six mice in each group. We did two independent experiments, each using three mice. Unpaired Student's *t*-tests were used. *p < 0.05, **p < 0.01, ***p < 0.001.

We tested if treatment with low-dose gemcitabine could enhance the ability of NK cells to kill tumor cells. First, we purified the splenic NK cells of wild-type C57BL/6 mice to kill untreated LLC cells and LLC cells treated with 10 nM gemcitabine for 24 h with an effector cell:target cell ratio of 1:1, 4:1 and 10:1 (Figure 5A). NK cells were more effective in killing efficiency in LLC cells treated with chemotherapy drugs (Figure 5A). Second, we established a model of LLC subcutaneous tumors in mice and treated them with gemcitabine (four times every 3 days) at low, medium and high doses plus cisplatin (twice every 6 days), with the PBS group as the control. After all treatments, the splenic NK cells of mice were purified to kill tumor cells with an effector cell:target cell ratio of 10:1 and 20:1 (Figures 5B–E). NK-cell cytotoxicity was enhanced in gemcitabine-treated groups relative to that in the control group (Figures 5C–E). NK-cell cytotoxicity was stronger in the low-dose gemcitabine group than that in the other two groups (Figures 5C–E). Also, we found that gemcitabine had no direct significant effects on expression of Ki67, NKG2D, or IFN-γ of NK cells (Supplementary Figures 4A,B). These data suggested that low-dose gemcitabine treated lung cancer cells are more sensitive to NK cell killing.

### Low-Dose Gemcitabine Treatment Is Sufficient to Inhibit the Growth of Lung Cancer Cells in Mice

**Figure 6:**
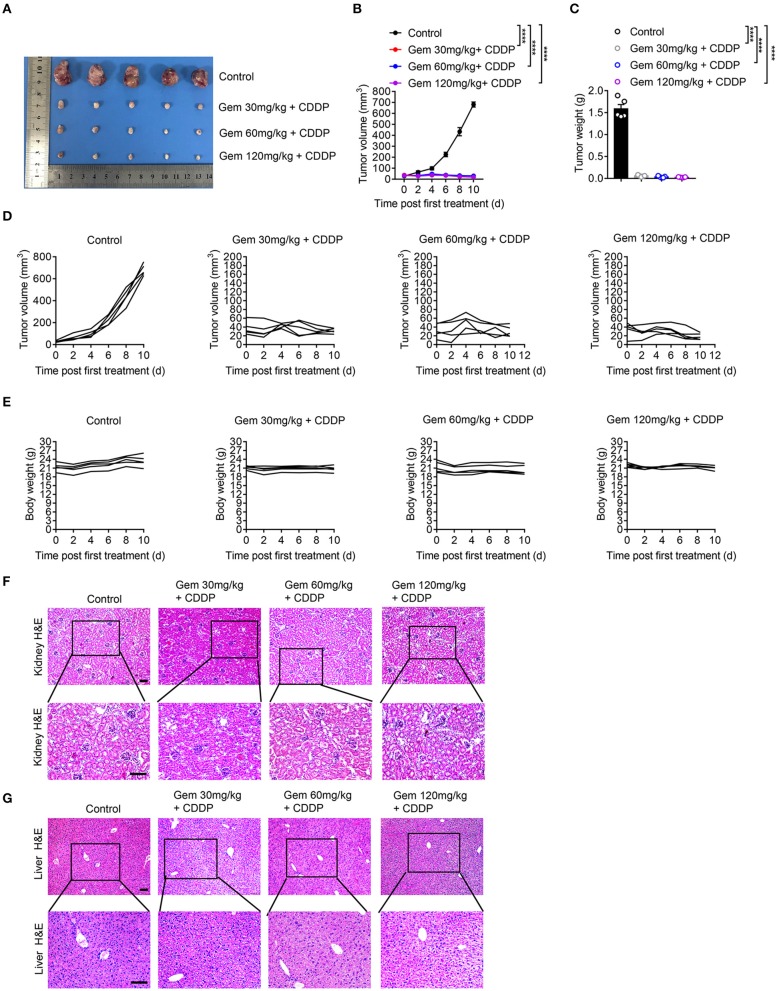
Low-dose gemcitabine treatment inhibits the growth of lung cancer cells in mice. **(A)** Subcutaneous tumor of mice (treated with gemcitabine at 30, 60, or 120 mg/kg combined with 3 mg/kg cisplatin). **(B–E)** Tumor size and body weight were measured. Representative histology (H&E staining) of kidneys **(F)** and livers **(G)** of mice after chemotherapy. Scale bar = 100 μm. Data are representative of three independent experiments. One-way and two-way analysis of variance (ANOVA) were used. ****p < 0.0001.

We established a model of LLC subcutaneous tumors in mice. Tumor size and body weight were measured to evaluate the efficacy of different doses of chemotherapy drugs. Treatment with a low (30 mg/kg), medium (60 mg/kg), and high (120 mg/kg) dose of gemcitabine plus cisplatin (3 mg/kg) suppressed LLC tumor volume significantly compared with that in the PBS-treated group (Figures 6A–D). Gemcitabine at different doses had no effect on body weight (Figure 6E). H&E staining of the liver showed vacuolar degeneration with increasing gemcitabine dose, but excessive kidney damage was not observed (Figures 6F,G).

These data suggested that low-dose gemcitabine treatment was sufficient to inhibit the growth of lung tumors in immunocompetent mice.

## Discussion

We observed that low-dose gemcitabine treatment increased the exposure of CRT and HMGB1, upregulated expression of NKG2D ligands, activated NK cells, and enhanced antitumor immunity. Low-dose gemcitabine treatment was not toxic to the immune system and triggered antitumor effects by enhancing immunogenicity. These data may have implications for gemcitabine-based immunochemotherapy regimens for immunocompetent lung cancer patients.

Chemotherapy agents used to be considered detrimental to the immune system because of their myelosuppressive effect. However, there is accumulating evidence that chemotherapy can play an active role in enhancing antitumor immune responses and may aid immunotherapy by activating the immune system rather than suppressing it (31).

“Tumor immunogenicity” is defined as the ability of a tumor to induce an immune response that can prevent its growth (32). Tumor cells treated with chemotherapy or radiotherapy can express “danger” and “eat-me” signals on the cell surface (e.g., NKG2D ligands, CRT) or can release immunostimulatory factors (e.g., HMGB1) to stimulate immune effectors (9). These signals are DAMPs that have non-immunological functions within cells before their exposure or release (6, 7, 33). DAMPs do not necessarily originate from dead cells and can be secreted or exposed by living cells undergoing life-threatening stress (34). CRT is a “molecular chaperone” in endoplasmic reticula, and is a mediator of tumor immunogenicity crucial for antitumor immunity (8). Obeid et al. (10) reported that anthracyclines induce translocation of CRT to the cell surface, and identified CRT as a key feature to anticancer immune responses and a possible strategy for immunogenic chemotherapy. HMGB1 is a chromatin-binding protein and can act as a strong cytokine and attract various immune cells (8, 35–37). HMGB1 is identified as a DAMP because it is released passively by necrotic cells, but not by apoptotic cells (38). Suzuki et al. (23) showed that chemoradiotherapy induced tumor antigen-specific T-cell responses, and that high local levels of HMGB1 correlated with longer patient survival. CRT exposure and HMGB1 release after stress are related to the activation of DCs, which are the most efficient antigen-presenting cells to prime the response of T cells (39–41). NKG2D ligands show low expression from healthy cells (14) and, in general, it is believed that their expression is upregulated in stressed cells (42). The interaction between NKG2D and NKG2D ligands is a key event in the regulation of innate and adaptive immune responses. After cellular stress (e.g., viral infection, DNA damage), expression of NKG2D ligands is upregulated, which leads to the lysis of target cells *via* activation of NKG2D signaling (39–41).

Gemcitabine is a nucleoside analog and an immunoregulatory agent. It is first-line chemotherapy for lung cancer but, inevitably, almost all patients eventually develop disease progression after treatment. Gemcitabine has been reported to inhibit myeloid-derived suppressor cells (MDSCs) directly in mice (42), and increase human leukocyte antigen class-I (HLA-I) expression in tumor cells (43).

To investigate if gemcitabine induces CRT and HMGB1 exposure, we treated lung cancer cells with gemcitabine (5, 10, 50, 100, or 500 nM) *in vitro*. Murine and human lung cancer cells treated with gemcitabine caused CRT expression from membranes and HMGB1 exposure from nuclei *in vitro*. Also, the effect was stronger at a low dose than at a high dose, indicating that low-dose gemcitabine treatment can induce stronger CRT and HMGB1 exposure in tumor cells.

Furthermore, to get closer to the clinical therapeutic regimen, we designed a combination of gemcitabine and cisplatin *in vivo*. Clinically, there is no single regimen of cisplatin alone in the treatment of lung cancer. Gemcitabine combined with cisplatin is a standard first-line treatment of NSCLC. Cisplatin alone should no longer be considered as an appropriate control arm in randomized studies of patients with advanced or metastatic NSCLC (44). Hence, we treated tumor-bearing mice with gemcitabine combined with cisplatin to ascertain if there was a similar phenomenon *in vivo*. The clinical dose of gemcitabine is 1,250 mg/m^2^ (1). Mice receiving gemcitabine treatment were given the maximum tolerated dose of 120 mg/kg (a dose similar to the equivalent dose used in humans), medium dose of 60 mg/kg or low dose of 30 mg/kg four times every 3 days (42, 45, 46). Cisplatin was given at 3 mg/kg twice every 6 days (2).

A high dose of a chemotherapy drug kills more tumor cells, but it also inhibits immune cells and weakens immunogenicity. NK cells serve as the first line of defense for the immune system because they connect the innate immune system with the adaptive immune system. NK cells play critical roles in anti-tumor immunity (47). Multiple chemical agents have been shown to modulate immune responses, including induction of expression of NKG2D ligands. Khallouf et al. (48) reported that immunochemotherapy with 5-fluorouacil and IFN-α increased expression of major histocompatibility complex class-I and NKG2D ligands on murine pancreatic carcinoma (Panc02) cells, which could be useful for enhancing tumor immunogenicity. Soriani et al. (49) noted that myeloma cells treated with low doses of therapeutic agents (e.g., doxorubicin, melphalan, bortezomib) used commonly in patients with multiple myeloma up-regulated expression of DNAM-1 and NKG2D ligands. Some scholars have shown that antitumor immunity is increased by NK cells through activation of NKG2D. Engagement of NKG2D receptors triggers NK cell–mediated cytotoxicity and provides a costimulatory signal for CD8^+^ T cells (50). Diefenbach et al. (15) reported that tumor cells transduced with NKG2D ligands are rejected by NK cells and/or CD8^+^ T cells and induce protective immunity to tumor re-challenge. Some researchers (51) used NKG2D blocking experiments to study the relevance of the NKG2D system for the efficacy of temozolomide and irradiation in syngeneic orthotopic glioblastoma models. Administration of the anti-NKG2D antibody abrogated the survival benefit conferred by temozolomide and attenuated the irradiation-mediated survival benefit in SMA-560 glioma-bearing mice. These results suggest that NKG2D plays a positive role in anti-tumor immunity. We also found that the level of IFN-γ produced by NKG2D^+^ NK cells and NKG2D^−^ NK cells are different. NKG2D^−^ NK cells have a low IFN-γ level, while NKG2D^+^ NK cells have a high level. In our study, gemcitabine upregulated expression of NKG2D ligands on mRNA and protein levels in lung cancer cells from humans and mice. Gemcitabine had no direct significant effects on expression of Ki67, NKG2D, and IFN-γ of purified splenic NK cells *in vitro*. Low-dose gemcitabine treated lung cancer cells are more sensitive to NK cell killing. Hence, we further explored the effects of gemcitabine on the immune system, NK-cell activation, and side effects *in vivo*.

Chemotherapy kills rapidly growing tumor cells, but also inhibits the growth of hair cells, bone-marrow cells, and cells within the gastrointestinal tract. The manifestations are hair loss, neutropenia, emesis, diarrhea and other gastrointestinal reactions. In our study, the percentage and absolute number of neutrophils in mice treated with gemcitabine decreased, thereby indicating the inhibitory effect of gemcitabine on the bone marrow in mice. The absolute number of lymphocytes did not decrease but the percentage of lymphocytes increased, demonstrating that gemcitabine hampers the numbers of myeloid cells but not the number of lymphocytes gemcitabine. Furthermore, we investigated lymphocyte function. Low-dose gemcitabine treatment increased the percentage of NK cells and MFI of IFN-γ^+^ NK^+^ cells, and high-dose gemcitabine treatment reduced the MFI of Ki67^+^ NK^+^ cells. These results provide further evidence that NK cells can be activated by low-dose gemcitabine treatment and induce a strong killing response, and that proliferation of NK cells was inhibited markedly at a high dose of gemcitabine. NK cells in mice treated with low-dose gemcitabine showed stronger killing *in vitro* than that using a high dose, suggesting that activation of NK cells by gemcitabine was one of the anti-tumor mechanisms in addition to cytotoxicity. We observed a significant reduction in tumor size, less hepatorenal toxicity, and fewer side effects at a low dose than that at a high dose.

## Data Availability Statement

All datasets generated for this study are included in the article/Supplementary Material.

## Ethics Statement

The animal study was reviewed and approved by the Ethics Committee of the University of Science and Technology of China (Hefei, China).

## Author Contributions

XZha, XZhe, and LX conceived and designed the experiments. XZha, DW, and DJ carried out the experiments. XZha, XZhe, ZL, LJ, and JC analyzed the data. XZha wrote the manuscript. LX and XZhe designed the research, supervised the work, and revised the manuscript.

### Conflict of Interest

The authors declare that the research was conducted in the absence of any commercial or financial relationships that could be construed as a potential conflict of interest.

## Supplementary Material

Supplementary Figure 1 High-dose gemcitabine induces lung cancer cell death. **(A,B)** LLC cells (up) and A549 cells (down) were treated with vehicle control (DMSO), gemcitabine (Gem; 5, 10, 50, 100, 500 nM), mitoxantrone (Mtx; 1 μM) or cisplatin (CDDP; 5 μM) at 37°C in a 5% CO_2_ incubator. Cells were stained with DEAD staining (R37601, Invitrogen™) for 15 min. A multi-photon confocal microscope (880 Meta; Zeiss, Weltzlar, Germany) was used to measure immunofluorescence. Red fluorescence is an indication of dead cells. Scale bar = 50 μm.

Supplementary Figure 2 The gating strategy and representative FACS profiles of subcutaneous tumor-burdened mice splenic lymphocytes. **(A,B)** Gating strategy and representative flow spots of NK cells (CD45^+^CD3^−^ CD19^−^NK1.1^+^), B cells (CD45^+^CD3^−^NK1.1^−^CD19^+^), CD4^+^ T cells (CD45^+^CD19^−^NK1.1^−^CD3^+^CD8^−^CD4^+^), and CD8^+^ T cells (CD45^+^CD19^−^ NK1.1^−^CD3^+^CD8^+^CD4^−^). Numbers adjacent to the outlined areas indicate the proportion of cells (%). **(C)** Expression of NKG2D, IFN-γ, and Ki67 in NK cells, detected by flow cytometry in gated NK cells (CD45^+^CD3^−^ CD19^−^NK1.1^+^). **(D)** Gating strategy and representative flow spots of NKG2D^+^ of NK cells (CD45^+^CD3^−^CD19^−^NK1.1^+^). Numbers adjacent to the outlined areas indicate the proportion (%) of cells.

Supplementary Figure 3 The levels of IFN-γ produced by NKG2D^+^ NK cells are higher than NKG2D^−^ NK cells. **(A,B)** The mice lymphocytes were freshly isolated. For IFN-γ staining, mice lymphocytes were incubated with phorbol myristate acetate (50 ng/mL), monensin (10 μg/mL) and ionomycin (1 μg/mL) for 4 h at 37°C in a 5% CO_2_ incubator. Then, lymphocytes were stained with extracellular antibodies (APC-CY7-labeled CD45.2, BV605-labeled -CD3, PE-CY7-labeled NK1.1, APC-labeled NKG2D) for 30 min at 4°C. After fixation and permeabilization, lymphocytes were stained with BV421-labeled IFN-γ for 30 min at 4°C. Mean fluorescence intensity (MFI) **(A)** and proportion **(B)** of IFN-γ of splenic NKG2D^+^ and NKG2D^−^ NK cells, detected by flow cytometry. Unpaired Student's *t*-tests were used. ^*^*p* < 0.05, ^**^*p* < 0.01.

Supplementary Figure 4 Gemcitabine has no direct significant effects on expression of Ki67, NKG2D, and IFN-γ of C57BL/6 splenic NK cells *in vitro*. **(A,B)** C57BL/6 mice lymphocytes **(A)** or purified NK cells **(B)** were freshly isolated and stimulated with 50 or 500 nM gemcitabine for 24 h at 37°C in a 5% CO_2_ incubator. MFI of splenic Ki67^+^ NK^+^ cells, IFN-γ^+^ NK^+^ cells, and NKG2D^+^ NK cells, detected by flow cytometry. One-way analysis of variance (ANOVA) was used.

Supplementary Figure 5 Low-dose gemcitabine treatment increases expression of IFN-γ while high-dose gemcitabine treatment impairs NK-cell proliferation. The proportion (%) of splenic IFN-γ^+^ and Ki67^+^ NK cells, detected by flow cytometry in gated NK cells (CD45^+^CD3^−^ CD19^−^NK1.1^+^). One-way analysis of variance (ANOVA) was used. ^*^*p* < 0.05.
