# Supplementary figures and images for: Low-Dose Gemcitabine Treatment Enhances Immunogenicity and Natural Killer Cell-Driven Tumor Immunity in Lung Cancer
Source: Front Immunol. 2020 Feb 25;11:331. doi: 10.3389/fimmu.2020.00331 (PMC7052388; doi:10.3389/fimmu.2020.00331)

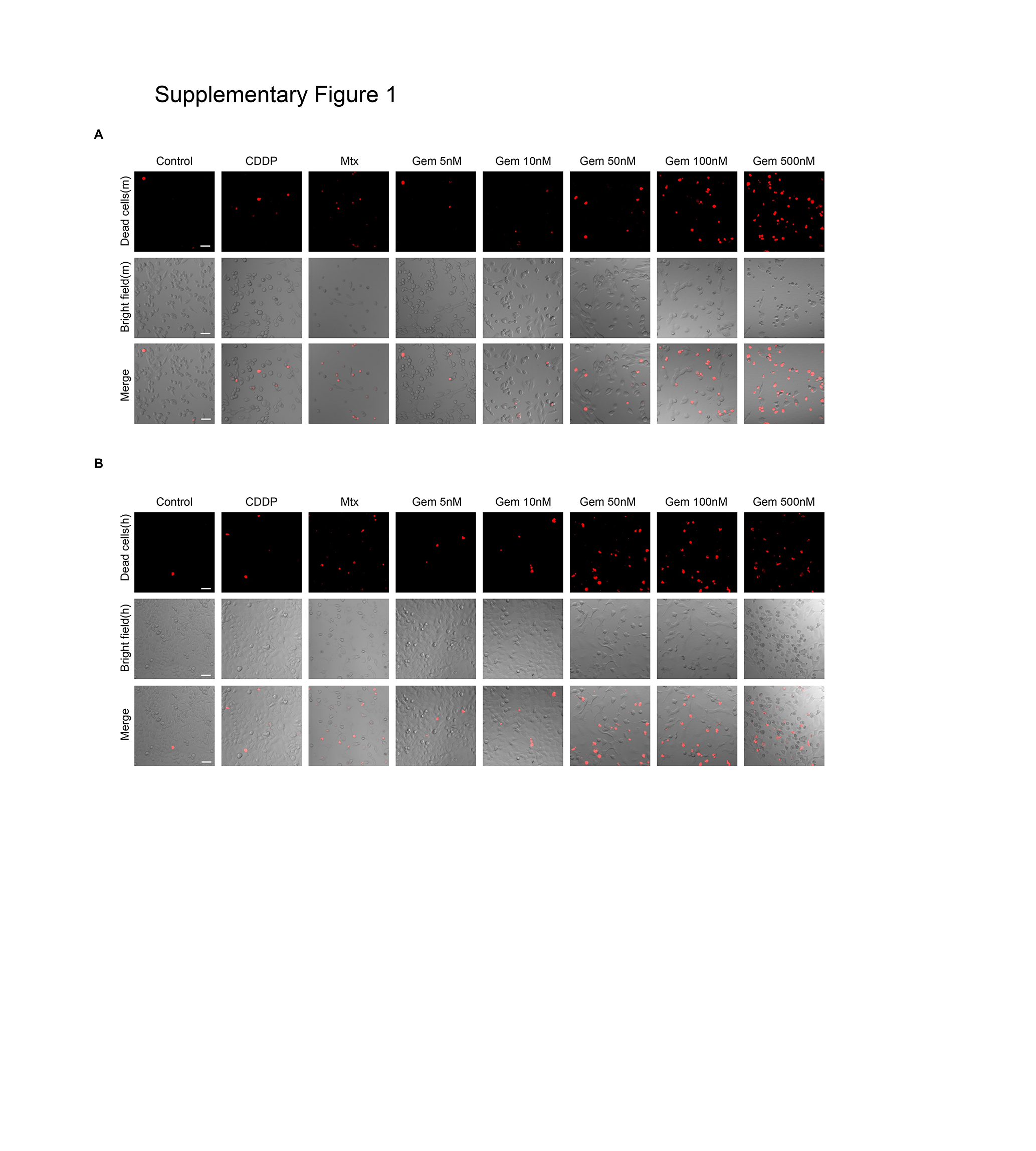

Supplement: Supplementary Figure 1 — High-dose gemcitabine induces lung cancer cell death. (A,B) LLC cells (up) and A549 cells (down) were treated with vehicle control (DMSO), gemcitabine (Gem; 5, 10, 50, 100, 500 nM), mitoxantrone (Mtx; 1 μM) or cisplatin (CDDP; 5 μM) at 37°C in a 5% CO2 incubator. Cells were stained with DEAD staining (R37601, Invitrogen™) for 15 min. A multi-photon confocal microscope (880 Meta; Zeiss, Weltzlar, Germany) was used to measure immunofluorescence. Red fluorescence is an indication of dead cells. Scale bar = 50 μm. [file Image_1.TIF]

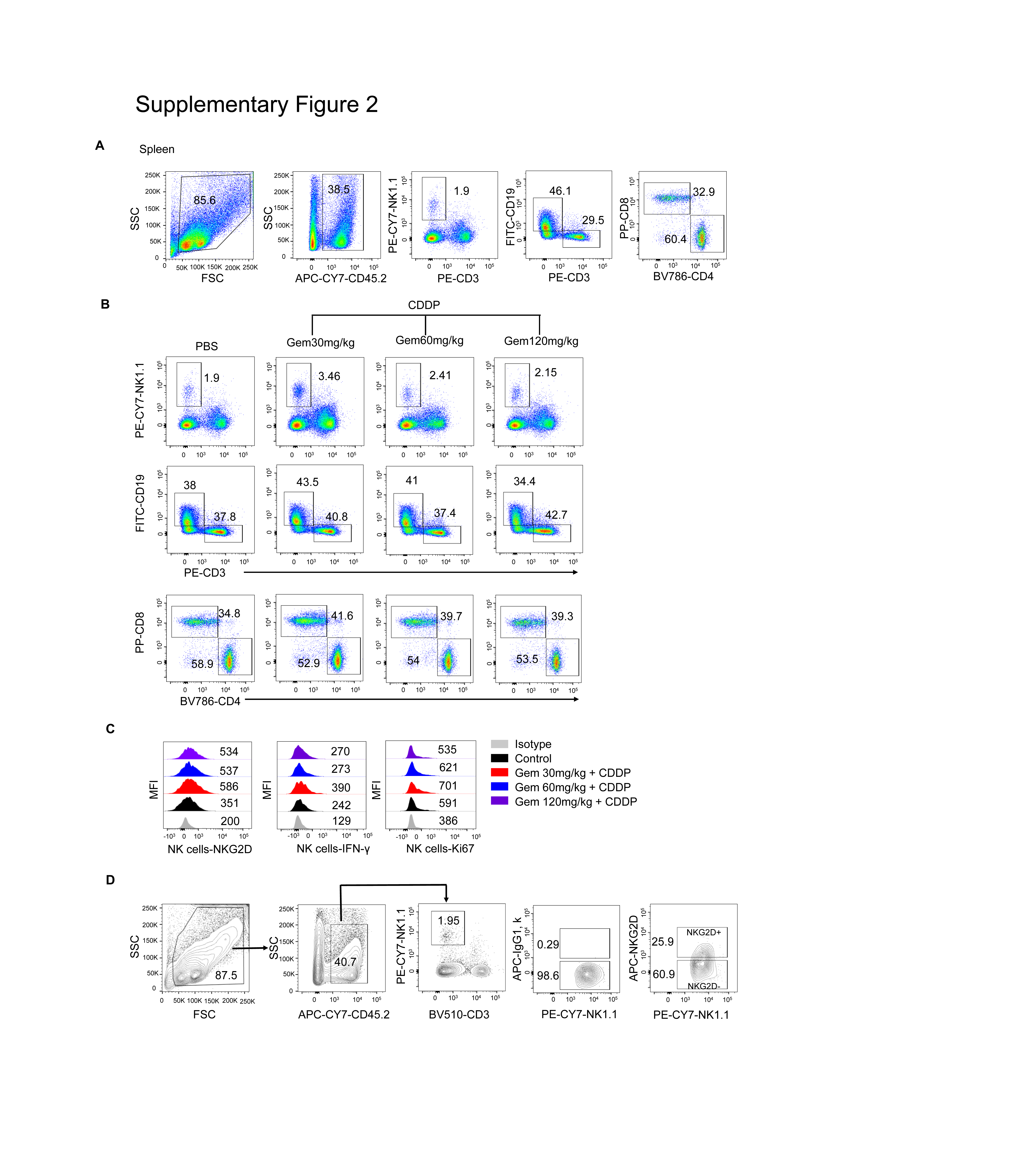

Supplement: Supplementary Figure 2 — The gating strategy and representative FACS profiles of subcutaneous tumor-burdened mice splenic lymphocytes. (A,B) Gating strategy and representative flow spots of NK cells (CD45+CD3− CD19−NK1.1+), B cells (CD45+CD3−NK1.1−CD19+), CD4+ T cells (CD45+CD19−NK1.1−CD3+CD8−CD4+), and CD8+ T cells (CD45+CD19− NK1.1−CD3+CD8+CD4−). Numbers adjacent to the outlined areas indicate the proportion of cells (%). (C) Expression of NKG2D, IFN-γ, and Ki67 in NK cells, detected by flow cytometry in gated NK cells (CD45+CD3− CD19−NK1.1+). (D) Gating strategy and representative flow spots of NKG2D+ of NK cells (CD45+CD3−CD19−NK1.1+). Numbers adjacent to the outlined areas indicate the proportion (%) of cells. [file Image_2.TIF]

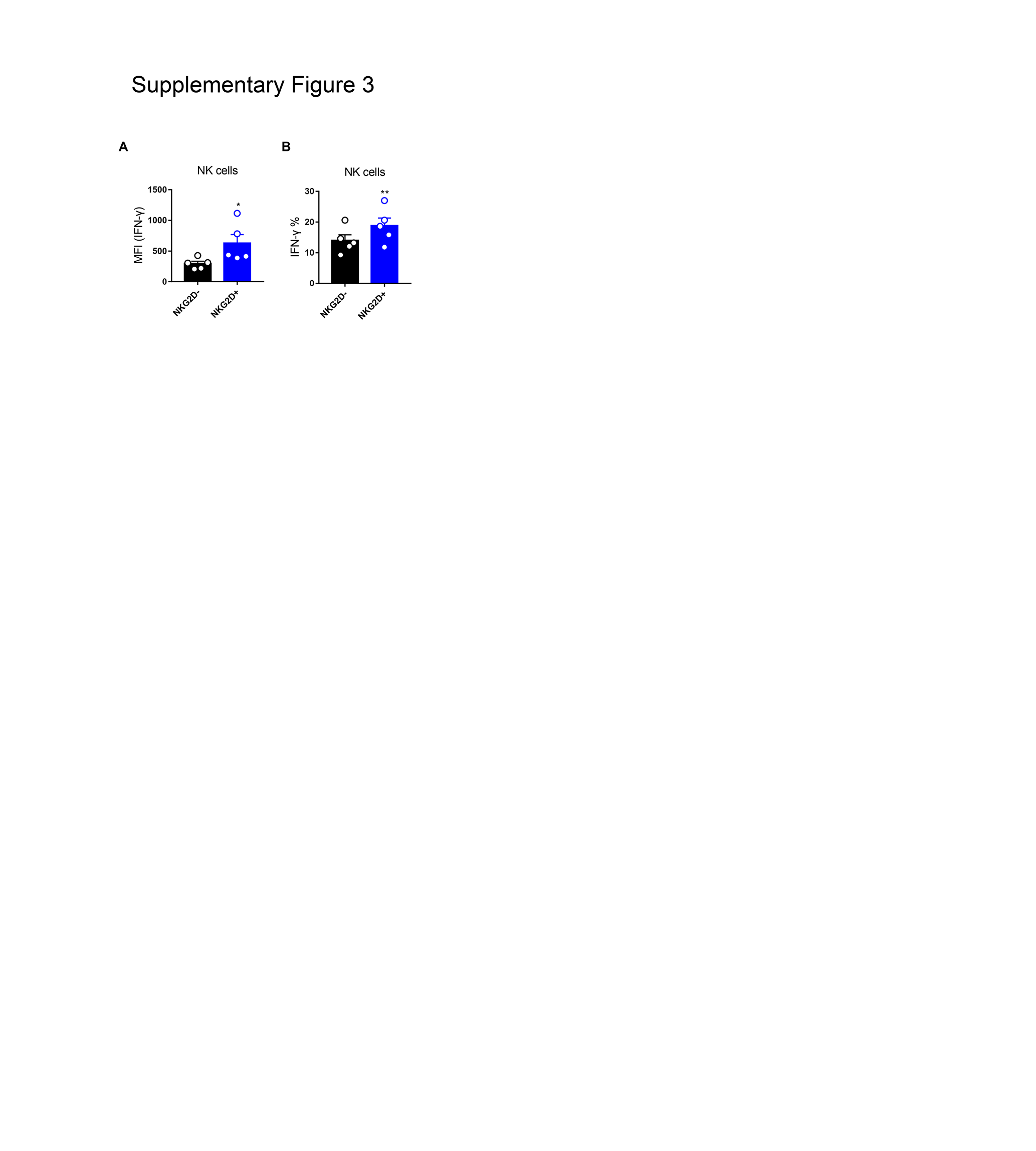

Supplement: Supplementary Figure 3 — The levels of IFN-γ produced by NKG2D+ NK cells are higher than NKG2D− NK cells. (A,B) The mice lymphocytes were freshly isolated. For IFN-γ staining, mice lymphocytes were incubated with phorbol myristate acetate (50 ng/mL), monensin (10 μg/mL) and ionomycin (1 μg/mL) for 4 h at 37°C in a 5% CO2 incubator. Then, lymphocytes were stained with extracellular antibodies (APC-CY7-labeled CD45.2, BV605-labeled -CD3, PE-CY7-labeled NK1.1, APC-labeled NKG2D) for 30 min at 4°C. After fixation and permeabilization, lymphocytes were stained with BV421-labeled IFN-γ for 30 min at 4°C. Mean fluorescence intensity (MFI) (A) and proportion (B) of IFN-γ of splenic NKG2D+ and NKG2D− NK cells, detected by flow cytometry. Unpaired Student's t-tests were used. *p < 0.05, **p < 0.01. [file Image_3.TIF]

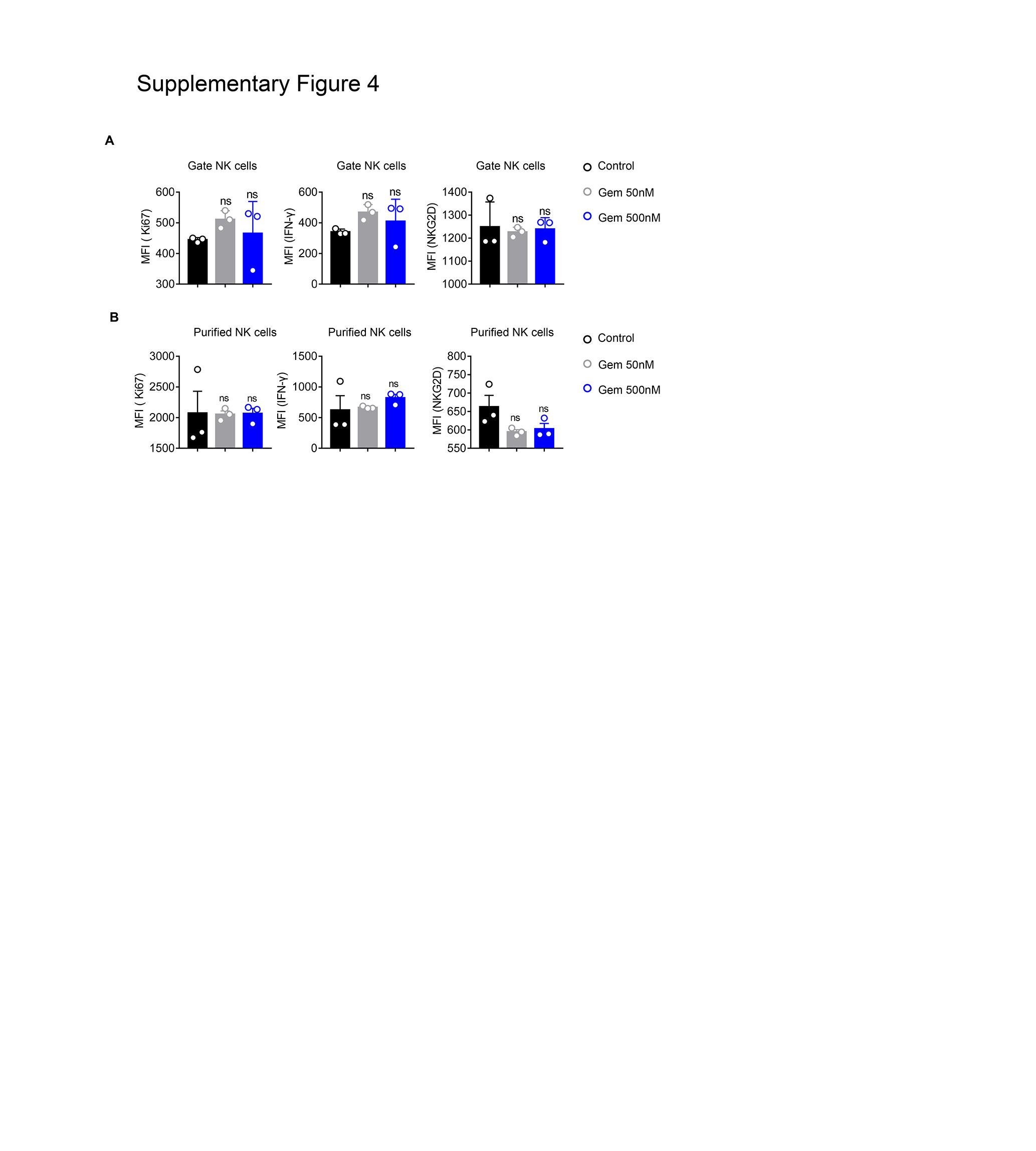

Supplement: Supplementary Figure 4 — Gemcitabine has no direct significant effects on expression of Ki67, NKG2D, and IFN-γ of C57BL/6 splenic NK cells in vitro. (A,B) C57BL/6 mice lymphocytes (A) or purified NK cells (B) were freshly isolated and stimulated with 50 or 500 nM gemcitabine for 24 h at 37°C in a 5% CO2 incubator. MFI of splenic Ki67+ NK+ cells, IFN-γ+ NK+ cells, and NKG2D+ NK cells, detected by flow cytometry. One-way analysis of variance (ANOVA) was used. [file Image_4.TIF]

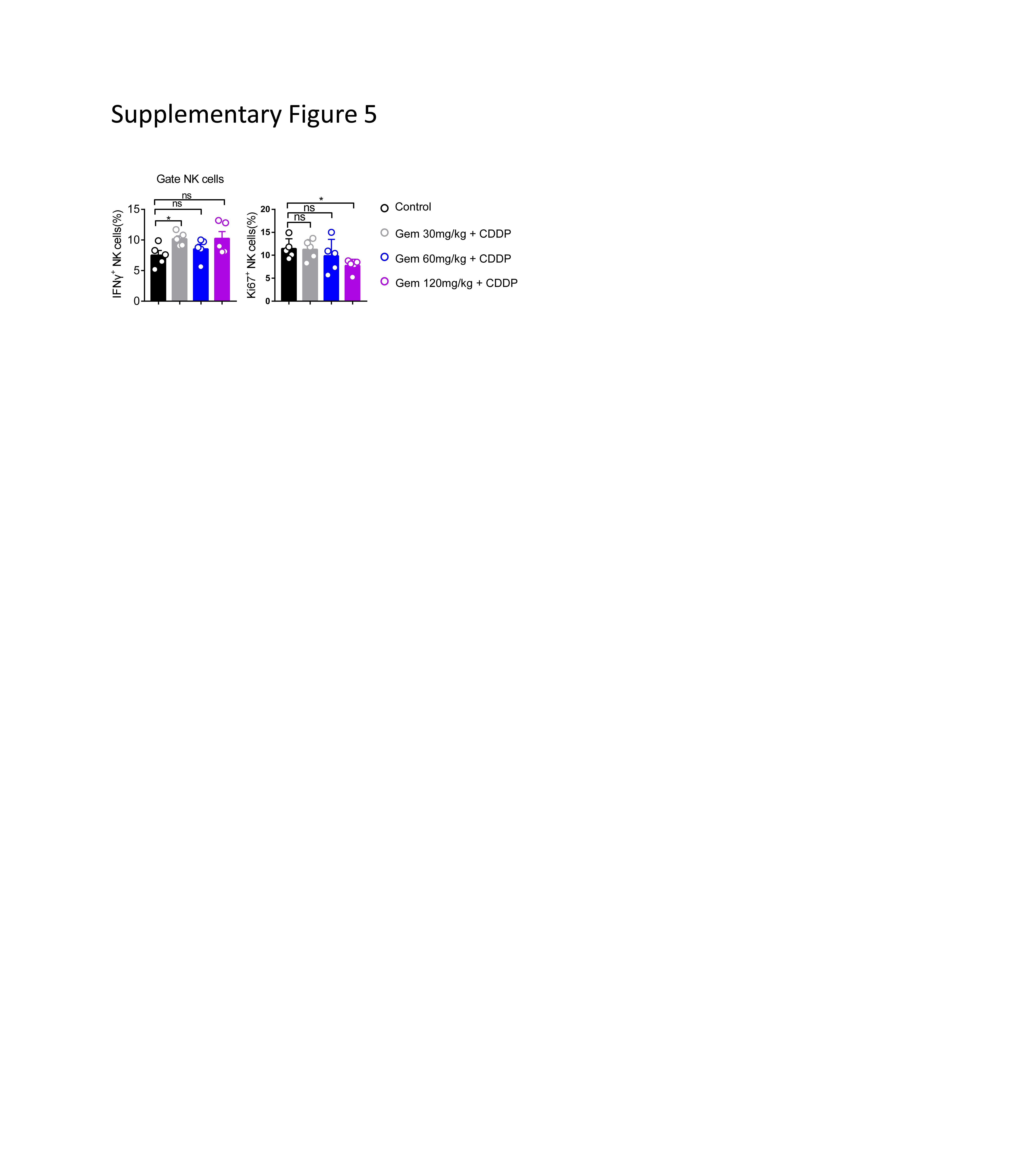

Supplement: Supplementary Figure 5 — Low-dose gemcitabine treatment increases expression of IFN-γ while high-dose gemcitabine treatment impairs NK-cell proliferation. The proportion (%) of splenic IFN-γ+ and Ki67+ NK cells, detected by flow cytometry in gated NK cells (CD45+CD3− CD19−NK1.1+). One-way analysis of variance (ANOVA) was used. *p < 0.05. [file Image_5.TIF]
